# Draft Genome Sequence of *Microbacterium oryzae* Strain MB-10, Isolated from a Rice Field in India

**DOI:** 10.1128/MRA.01532-19

**Published:** 2020-02-06

**Authors:** Sushanta Deb, Subrata K. Das

**Affiliations:** a Department of Biotechnology, Institute of Life Sciences, Bhubaneswar, India; University of Maryland School of Medicine

## Abstract

We report the draft genome sequence of Microbacterium oryzae strain MB-10^T^, which was isolated from rice field soil. The genome is 3.04 Mb, with a G+C content of 71.10%, and encodes 2,727 proteins. The genome sequence provides further information about strain MB-10^T^ and the genus *Microbacterium*.

## ANNOUNCEMENT

The genus *Microbacterium* was first proposed by Orla-Jensen (1) and was amended by Collins et al. (2). Takeuchi and Hatano united the genera *Microbacterium* and *Aureobacterium* in the single genus *Microbacterium*, based on the phenotypic and phylogenetic relatedness of the genera (3). To date, 101 recognized species have been validly published under the genus *Microbacterium* (http://www.bacterio.net/microbacterium.html). Gneiding et al. reported the frequent presence of *Microbacterium* spp. in human clinical specimens, and thus they are considered opportunistic human pathogens (4). A recent study demonstrated multidrug resistance in pathogenic *Microbacterium* spp. isolated from a cystic fibrosis patient (5).

Microbacterium oryzae was reported by Kumari et al. (6) and was isolated from rice field soil samples collected from Jagatpur, Odisha, India (coordinates, 20.49694 N, 85.94444 E). Strain MB-10 was grown at 28°C in nutrient broth (Difco, USA), as described earlier (6). Genomic DNA was isolated using the QIAamp DNA minikit (Qiagen, Germany). The quality (*A*_260_/*A*_280_ ratio) and concentration of DNA were determined using a NanoDrop 8000 UV-visible spectrophotometer and a Qubit 2.0 fluorometer (Thermo Fisher Scientific, USA). DNA was sheared using a g-TUBE device, according to the manufacturer’s protocol (Covaris, Woburn, MA, USA). Fragmented DNA with an average length of 10 kb was used for SMRTbell library preparation, as recommended by the manufacturer. The quantity and quality of the SMRTbell libraries were evaluated using a high-sensitivity double-stranded DNA kit with a Qubit fluorometer and a DNA 12000 kit with a 2100 Bioanalyzer (Agilent, Santa Clara, CA, USA), respectively. Sequencing was performed with the PacBio Sequel sequencing system (Pacific Biosciences, USA).

Quality control of the sequencing reads was performed using the parameters correct and trim in Canu 1.3. *De novo* genome assembly of PacBio reads was performed with the Canu 1.3 assembler (https://github.com/marbl/canu) (parameters: correct; p, bacteria; merylMemory, 15; batThreads, 12; stopOnLowCoverage, 100; genomeSize, 3.0m) (7). The scaffolding was performed using the Single Molecular Integrative Scaffolding (SMIS) pipeline (https://github.com/fg6/smis) (parameters: score, 50; len, 2000; step, 200; contig, 3000; edge, 5). Finally, the gaps were filled with the help of PBJelly (parameters: minMatch, 8; minPctIdentity, 70; bestn, 1; nCandidates, 10; maxScore, 500; nproc, 8; noSplitSubreads) (8). A Perl script (https://github.com/tomdeman-bio/Sequence-scripts/blob/master/calc_N50_GC_genomesize.pl) was used to calculate the genome size and the G+C content of the assembled genome. A total of 375,722 PacBio reads were assembled, which generated a single scaffold with input read sequencing depth of 366×. The raw read *N*_50_ value was 4,213 bp. The draft genome was annotated using the NCBI Prokaryotic Genome Annotation Pipeline (PGAP 4.9) (9). The genome is 3.04 Mb, with a G+C content of 71.10%. The final draft genome contains 2,727 protein-coding sequences, 47 tRNAs, 3 5S rRNAs, 3 16S rRNAs, and 3 23S rRNAs.

Comparative genomic analysis was performed with the genome sequences of type strains available in the NCBI database, to evaluate the genomic relatedness, using JSpeciesWS (10). The average nucleotide identities between Microbacterium oryzae strain MB-10^T^ and the reference genomes were <96%, indicating a type species (11). Further, *in silico* DNA-DNA hybridization (DDH) was performed with the reference strains to calculate the genome-to-genome distances (12). *In silico* DDH values were 20.30% to 20.40%, which are below the threshold value of 70%, justifying a type species. The type strain is MB-10^T^ (also designated JCM 16837^T^ or DSM 23396^T^).

### Data availability.

The whole-genome shotgun sequence of Microbacterium oryzae strain MB-10^T^ has been deposited in DDBJ/ENA/GenBank under accession number CP032550. SRA data are available in the NCBI SRA database under accession number SRR7825136.
